# Monte Carlo‐based beam quality and phantom scatter corrections for solid‐state detectors in ${\;}^{60}\text{Co}$ and ${\;}^{192}\text{Ir}$ brachytherapy dosimetry

**DOI:** 10.1120/jacmp.v15i6.4907

**Published:** 2014-11-08

**Authors:** Mishra Subhalaxmi, T. Palani Selvam

**Affiliations:** ^1^ Radiological Physics & Advisory Division Health, Safety & Environment Group, Bhabha Atomic Research Centre Mumbai 400 094 Maharashtra India

**Keywords:** Monte Carlo, brachytherapy, beam quality correction, phantom scatter correction

## Abstract

Beam quality correction, $k_{{\text{QQ}}_{0}}(r)$, for solid‐state detectors diamond, LiF, ${\text{Li}}_{2}B_{4}O_{7},{\text{Al}}_{2}O_{3}$, and plastic scintillator are calculated as a function of distance, r, along the transverse axis of the ${\;}^{60}\text{Co}$ and ${\;}^{192}\text{Ir}$ brachytherapy sources using the Monte Carlo‐based EGSnrc code system. This study also includes calculation of detector‐specific phantom scatter correction, $k_{\text{phan}}(r)$, for solid phantoms such as PMMA, polystyrene, solid water, virtual water, plastic water, RW1, RW3, A150, and WE210. For ${\;}^{60}\text{Co}$ source, $k_{{\text{QQ}}_{0}}(r)$ is about unity and distance‐independent for diamond, plastic scintillator, ${\text{Li}}_{2}B_{4}O_{7}$ and LiF detectors. For this source, $k_{{\text{QQ}}_{0}}(r)$ decreases gradually with r for ${\text{Al}}_{2}O_{3}$ detector (about 6% smaller than unity at 15 cm). For ${\;}^{192}\text{Ir}$ source, $k_{{\text{QQ}}_{0}}(r)$ is about unity and distance‐independent for ${\text{Li}}_{2}B_{4}O_{7}$ detector (overall variation is about 1% in the distance range of 1–15 cm). For this source, $k_{{\text{QQ}}_{0}}(r)$ increases with r for diamond and plastic scintillator (about 6% and 8% larger than unity at 15 cm, respectively). Whereas $k_{{\text{QQ}}_{0}}(r)$ decreases with r gradually for LiF (about 4% smaller than unity at 15 cm) and steeply for ${\text{Al}}_{2}O_{3}$ (about 25% smaller than unity at 15 cm). For ${\;}^{60}\text{Co}$ source, solid water, virtual water, RW1, RW3, and WE210 phantoms are water‐equivalent for all the investigated solid‐state detectors. Whereas polystyrene and plastic water phantoms are water‐equivalent for diamond, plastic scintillator, ${\text{Li}}_{2}B_{4}O_{7}$ and LiF detectors, but show distance‐dependent $k_{\text{phan}}(r)$ values for ${\text{Al}}_{2}O_{3}$ detector. PMMA phantom is water‐equivalent at all distances for ${\text{Al}}_{2}O_{3}$ detector, but shows distance‐dependent $k_{\text{phan}}(r)$ values for remaining detectors. A150 phantom shows distance‐dependent $k_{\text{phan}}(r)$ values for all the investigated detector materials. For ${\;}^{192}\text{Ir}$ source, solid water, virtual water, RW3, and WE210 phantoms are water‐equivalent for diamond, plastic scintillator, ${\text{Li}}_{2}B_{4}O_{7}$ and LiF detectors, but show distance‐dependent $k_{\text{phan}}(r)$ values for ${\text{Al}}_{2}O_{3}$ detector. All other phantoms show distance‐dependent $k_{\text{phan}}(r)$ values for all the detector materials.

PACS numbers: 87.10.Rt, 87.53.Bn, 87.53.Jw

## I. INTRODUCTION

${\;}^{192}\text{Ir}$ and ${\;}^{60}\text{Co}$ sources are used in high‐dose‐rate (HDR) brachytherapy.^1^, ^2^, ^3^, ^4^ Dosimetry of a brachytherapy source is generally carried out using various solid‐state detectors. The response of the detector is required to be corrected for absorbed dose energy dependence, when it is not water‐equivalent. Although water is recommended as the reference medium for dosimetry of brachytherapy sources,^5^, ^6^ different solid phantoms are also used to overcome practical problems such as waterproofing and precise positioning of detectors. In a previously published article,^(7)^ relative absorbed‐dose energy response corrections, R, were reported for different solid‐state detectors for ${\;}^{169}\text{Yb}$ and ${\;}^{125}I$ brachytherapy sources. The study also included the influence of solid phantom materials such as PMMA (polymethylmethacrylate) and polystyrene on R. In another study,^(8)^ the values of R were reported for different radiochromic films for high‐energy brachytherapy sources such as ${\;}^{60}\text{Co},{\;}^{137}\text{Cs},{\;}^{192}\text{Ir}$, and ${\;}^{169}\text{Yb}$ in liquid water, PMMA, and polystyrene phantom materials. In a recently published article by Selvam et al.,^(9)^ beam quality corrections (which is the inverse of R) were reported for different solid‐state detectors at ${\;}^{137}\text{Cs}$ energy. In addition, detector‐specific phantom scatter corrections for different solid phantoms were also reported in their study. The purpose of this study is to calculate the beam quality corrections for solid‐state detectors for ${\;}^{60}\text{Co}$ and ${\;}^{192}\text{Ir}$ brachytherapy sources. This study also includes detector‐specific phantom scatter corrections for different solid phantoms for these sources. The investigation of phantom scatter also includes water as a detector material. The EGSnrc‐based^(10)^ user‐codes DOSRZnrc and FLURZnrc ^(11)^ are used in the study.

## II. MATERIALS AND METHODS

### A. Radioactive sources

The brachytherapy sources investigated in this study are BEBIG HDR ${\;}^{60}\text{Co}$ (model Co0.A86; Eckert & Zielger BEBIG BmbH, Berlin, Germany)^(3)^ and HDR ${\;}^{192}\text{Ir}$ (model MicroSelectron; Elekta, Stockholm, Sweden).^(2)^ In the Monte Carlo calculations, two gamma lines 1.17 MeV and 1.33 MeV are considered for the ${\;}^{60}\text{Co}$ source. For ${\;}^{192}\text{Ir}$ source, the spectrum is taken from the literature.^(12)^

### B. Detector and phantom materials

The detector materials investigated in this study are diamond, LiF, ${\text{Li}}_{2}B_{4}O_{7}$, plastic scintillator, and ${\text{Al}}_{2}O_{3}$. The solid phantom materials investigated are PMMA, polystyrene, solid water, virtual water, plastic water, RW1, RW3, A150, and WE210. The atomic composition and density details of RW1 and virtual water phantoms are taken from the published studies.^13^, ^14^ Remaining phantom data are taken from the study by Seco and Evans^(15)^

### C. Beam quality and phantom scatter corrections

As described in the published study by Selvam et al.,^(9)^ beam quality correction, $k_{{\text{QQ}}_{0}}(r)$, and phantom scatter correction, $k_{\text{phan}}(r)$, can be calculated at a brachytherapy beam quality Q for solid‐state detectors by using the following relations:

(1) $$k_{QQ_{0}}(r)=\frac{\left[D_{w,Q}(r)/D_{\text{det},Q}(r)\right]}{\left[D_{w,Q_{0}}/D_{\text{det},Q_{0}}\right]}$$

(2) $$k_{\text{phan}}(r)=\left[D_{\text{det},Q}(r)/D_{\text{det},\text{phan},Q}(r)\right]$$

Here, $D_{w,Q}(r)$ and $D_{\text{det},Q}(r)$ are the absorbed dose to water and absorbed dose to detector in liquid water at a distance, r, along the transverse axis of the photon emitting brachytherapy source of beam quality Q (in the present study, it is ${\;}^{192}\text{Ir}$ or ${\;}^{60}\text{Co}$), respectively. $D_{w,Q}(r)$ and $D_{\text{det},Q}(r)$ are the absorbed dose to water and absorbed dose to detector in water at the reference beam quality $Q_{0}$ (realistic ${\;}^{60}\text{Co}$ teletherapy beam), respectively. $D_{\text{det},\text{phan},Q}(r)$ is the absorbed dose to detector in the solid phantom at r from the brachytherapy source of beam quality Q.

For the calculation of $k_{{\text{QQ}}_{0}}(r)$, the values of water‐to‐detector dose ratio at $Q_{0}$ (denominator of Eq. (1)) are taken from the published article.^(7)^ Note that $k_{\text{phan}}(r)$ converts absorbed dose to detector at r for the brachytherapy source (of beam quality Q) in a solid phantom to absorbed dose to detector in liquid water phantom at the same r. Numerator of $k_{{\text{QQ}}_{0}}(r)$ corrects for the difference in the energy absorption properties of water and detector at brachytherapy beam quality Q at r, and the denominator of $k_{{\text{QQ}}_{0}}(r)$ corrects for the same, but at reference beam quality $Q_{0}$.

### D. Monte Carlo calculations

Dose ratios of water‐to‐detector at beam quality Q (numerator of Eq. (1)) are based on the FLURZnrc user‐code^(11)^ as described in the published study.^7^, ^8^, ^9^ In the Monte Carlo calculations, the source is positioned at the centre of a 40 cm diameter ×40cm height cylindrical phantoms (liquid water and solid phantoms). The photon fluence spectrum is scored along the transverse axis of the source (r=1−15cm) in 0.5 mm high and 0.5 mm thick cylindrical shells. The fluence spectrum is converted to collision kerma‐to‐water and collision kerma‐to‐detector materials by using the mass‐energy absorption coefficients of water and detector materials, respectively.^(16)^ Note that the FLURZnrc^(11)^ simulations also provide fluence‐weighted mean energy of photons, $E_{\text{fl}}$. Up to 10^9^ photon histories are simulated. The 1 σ statistical uncertainty on the calculated absorbed dose and collision kerma values are about 0.2%. The statistical uncertainties on the calculated values of $k_{{\text{QQ}}_{0}}(r)$ and $k_{\text{phan}}(r)$ are less than 0.5%. The Monte Carlo parameters used in the calculations are same as that used in the earlier work.^8^, ^9^

## III. RESULTS & DISCUSSION

### A. Fluence‐weighted mean energy, $E_{\text{fl}}$

Tables 1, 2 present the values of $E_{\text{fl}}$ as a function of r for ${\;}^{60}\text{Co}$ and ${\;}^{192}\text{Ir}$ sources in various phantoms, respectively. $E_{\text{fl}}$ decreases with distance due to degradation in the photon energy after scattering. The degree of decrease depends on the type of the phantom as well as the type of source. For the ${\;}^{60}\text{Co}$ source, the decrease in $E_{\text{fl}}$ is higher in PMMA, polystyrene, and A150 phantom as compared to other phantoms. For example, $E_{\text{fl}}$ decreases from 1.134 MeV to 455 keV in PMMA, from 1.146 MeV to 486 keV in polystyrene, and from 1.140 MeV to 481 keV in A150 phantom when the distance is increased from 1 cm to 15 cm. For phantoms such as water, RW1, RW3, and solid water, $E_{\text{fl}}$ decreases from about 1.15 MeV to 520 keV in the above distance range. For the virtual water and WE210 phantoms, $E_{\text{fl}}$ decreases from about 1.150 MeV to 530 keV and, in the case of plastic water, from 1.152 MeV to 562 keV in the above distance range.

**Table 1:** Monte Carlo‐calculated values of fluence‐weighted mean energy for different phantoms presented as a function of distance r along the transverse axis of the BEBIG ${\;}^{60}\text{Co}$ source.

*Distance, r (cm)*	*Water*	*PMMA*	*Polystyrene*	*Plastic water*	*RW1*	*RW3*	*Virtual water*	*Solid water*	*A150*	*WE210*
1	1.149	1.134	1.146	1.152	1.151	1.149	1.150	1.149	1.140	1.152
2	1.057	1.026	1.049	1.063	1.061	1.056	1.058	1.058	1.036	1.061
3	0.972	0.927	0.958	0.982	0.974	0.969	0.973	0.972	0.943	0.980
4	0.896	0.842	0.877	0.913	0.900	0.891	0.899	0.897	0.861	0.905
5	0.832	0.770	0.807	0.851	0.831	0.824	0.835	0.830	0.792	0.841
6	0.774	0.709	0.746	0.796	0.774	0.766	0.778	0.776	0.731	0.785
7	0.723	0.656	0.693	0.751	0.726	0.715	0.728	0.726	0.681	0.735
8	0.682	0.612	0.649	0.712	0.683	0.672	0.687	0.684	0.637	0.694
9	0.644	0.575	0.608	0.678	0.646	0.636	0.650	0.648	0.602	0.659
10	0.612	0.546	0.576	0.648	0.614	0.603	0.620	0.615	0.569	0.626
11	0.585	0.517	0.550	0.623	0.587	0.577	0.594	0.589	0.543	0.599
12	0.563	0.495	0.528	0.603	0.564	0.556	0.571	0.567	0.522	0.578
13	0.547	0.477	0.511	0.585	0.548	0.539	0.555	0.549	0.503	0.559
14	0.532	0.462	0.498	0.573	0.533	0.523	0.542	0.534	0.490	0.547
15	0.520	0.455	0.486	0.562	0.523	0.512	0.530	0.524	0.481	0.536

**Table 2:** Monte Carlo‐calculated values of fluence‐weighted mean energy for different phantoms presented as a function of distance r along the transverse axis of the ${\;}^{192}\text{Ir}$ source.

*Distance, r (cm)*	*Water*	*PMMA*	*Polystyrene*	*Plastic water*	*RW1*	*RW3*	*Virtual water*	*Solid water*	*A150*	*WE210*
1	0.325	0.320	0.324	0.327	0.325	0.324	0.325	0.325	0.321	0.326
2	0.295	0.285	0.292	0.299	0.295	0.294	0.296	0.294	0.288	0.296
3	0.270	0.257	0.265	0.276	0.270	0.269	0.271	0.270	0.262	0.272
4	0.249	0.234	0.242	0.258	0.249	0.247	0.250	0.250	0.240	0.252
5	0.233	0.216	0.223	0.242	0.232	0.229	0.233	0.233	0.223	0.235
6	0.218	0.200	0.208	0.230	0.217	0.215	0.220	0.219	0.208	0.221
7	0.206	0.188	0.195	0.220	0.205	0.203	0.208	0.207	0.196	0.210
8	0.197	0.178	0.184	0.210	0.195	0.193	0.198	0.197	0.186	0.200
9	0.189	0.169	0.175	0.203	0.187	0.185	0.190	0.189	0.178	0.192
10	0.182	0.162	0.168	0.196	0.179	0.177	0.184	0.183	0.171	0.186
11	0.175	0.156	0.161	0.192	0.173	0.171	0.178	0.176	0.165	0.179
12	0.170	0.152	0.156	0.186	0.168	0.166	0.172	0.172	0.160	0.174
13	0.166	0.147	0.152	0.183	0.164	0.162	0.169	0.168	0.156	0.171
14	0.163	0.145	0.148	0.180	0.161	0.159	0.165	0.165	0.153	0.167
15	0.161	0.142	0.147	0.178	0.159	0.156	0.163	0.163	0.151	0.165

For ${\;}^{192}\text{Ir}$ source, decrease in $E_{\text{fl}}$ is higher for PMMA, A150, and polystyrene phantoms as compared to other phantoms. $E_{\text{fl}}$ decreases from about 320 keV to 140 keV when the distance is increased from 1 cm to 15 cm. For phantoms such as water, WE210, virtual water, and solid water, $E_{\text{fl}}$ decreases from about 325 keV to 160 keV in the above distance range. For RW1 and RW3 phantoms, $E_{\text{fl}}$ decreases from about 325 keV to 156 keV in the above distance range. In the case of plastic water phantom, $E_{\text{fl}}$ decreases from 327 keV to 178 keV when the distance is increased from 1 cm to 15 cm.

### B. Beam quality correction, $k_{{\text{QQ}}_{0}}(r)$

Table 3 presents the values of $k_{{\text{QQ}}_{0}}(r)$ for the ${\;}^{60}\text{Co}$ and ${\;}^{192}\text{Ir}$ sources, respectively. For ${\text{Li}}_{2}B_{4}O_{7}$ detector, $k_{{\text{QQ}}_{0}}(r)$ is about unity, and is independent of r for both the sources. For the ${\;}^{60}\text{Co}$ source, $k_{{\text{QQ}}_{0}}(r)$ is about unity and distance independent for diamond, plastic scintillator, and LiF detectors. Whereas for the ${\;}^{192}\text{Ir}$ source, $k_{{\text{QQ}}_{0}}(r)$ increases gradually about 6% and 8% larger than unity for diamond and plastic scintillator, but decreases about 4% smaller than unity for LiF detector with r over the distance range of 1–15 cm. For ${\text{Al}}_{2}O_{3}$ detector, $k_{{\text{QQ}}_{0}}(r)$ decreases with r gradually about 6% and steeply about 25% smaller than unity for ${\;}^{60}\text{Co}$ and ${\;}^{192}\text{Ir}$ sources respectively, in the above distance range.

**Table 3:** Beam quality correction, $k_{QQ_{0}}(r)$, presented for diamond, ${\text{Al}}_{2}O_{3},{\text{Li}}_{2}B_{4}O_{7}$, LiF, and plastic scintillator detectors as a function of distance r along the transverse axis of ${\;}^{60}\text{Co}$ and ${\;}^{192}\text{Ir}$ sources.

*Distance, r (cm)*	*Diamond*		$Al_{2}O_{3}$		$Li_{2}B_{4}O_{7}$		*LiF*		*Plastic Scintillator*	
	${\;}^{60}\text{Co}$	${\;}^{192}\text{Ir}$	${\;}^{60}\text{Co}$	${\;}^{192}\text{Ir}$	${\;}^{60}\text{Co}$	${\;}^{192}\text{Ir}$	${\;}^{60}\text{Co}$	${\;}^{192}\text{Ir}$	${\;}^{60}\text{Co}$	${\;}^{192}\text{Ir}$
1	1.000	1.004	0.998	0.973	1.000	1.000	1.000	0.996	1.001	1.017
2	1.001	1.008	0.996	0.955	1.000	1.001	0.999	0.994	1.001	1.022
3	1.001	1.012	0.992	0.935	1.000	1.001	0.999	0.991	1.002	1.027
4	1.002	1.016	0.989	0.913	1.000	1.002	0.998	0.987	1.003	1.031
5	1.003	1.021	0.984	0.892	1.000	1.003	0.998	0.984	1.003	1.037
6	1.003	1.026	0.980	0.870	1.000	1.003	0.997	0.980	1.004	1.043
7	1.004	1.031	0.975	0.849	1.000	1.004	0.996	0.977	1.005	1.048
8	1.005	1.036	0.970	0.830	1.000	1.005	0.996	0.973	1.006	1.055
9	1.006	1.041	0.965	0.813	1.001	1.006	0.995	0.970	1.007	1.061
10	1.007	1.045	0.960	0.797	1.001	1.006	0.994	0.967	1.008	1.067
11	1.008	1.050	0.956	0.783	1.001	1.007	0.994	0.964	1.009	1.071
12	1.009	1.054	0.952	0.770	1.001	1.008	0.993	0.961	1.010	1.075
13	1.009	1.057	0.949	0.760	1.001	1.008	0.993	0.959	1.011	1.078
14	1.010	1.060	0.946	0.752	1.001	1.009	0.992	0.957	1.012	1.083
15	1.010	1.062	0.944	0.746	1.001	1.009	0.992	0.956	1.012	1.084

### C. Phantom scatter correction, $k_{\text{phan}}(r)$

Table 4 presents the summary of $k_{\text{phan}}(r)$ results for diamond, ${\text{Al}}_{2}O_{3},{\text{Li}}_{2}B_{4}O_{7}$, LiF, and plastic scintillator detectors in the investigated phantom materials for the ${\;}^{60}\text{Co}$ and ${\;}^{192}\text{Ir}$ sources, respectively. In this table, phantoms which are water‐equivalent (i.e., $k_{\text{phan}}(r)$ is unity) at all distances (1–15 cm) are designated as “Yes”. “No” implies that the phantoms show distance‐dependent $k_{\text{phan}}(r)$ values. For such phantoms results are discussed below.

**Table 4:** Summary of $k_{\text{phan}}(r)$ results presented for diamond, ${\text{Al}}_{2}O_{3},{\text{Li}}_{2}B_{4}O_{7}$, LiF, and plastic scintillator detectors for the ${\;}^{60}\text{Co}$ and ${\;}^{192}\text{Ir}$ sources, respectively. In this table, “Yes” implies the phantom is water‐equivalent (i.e., $k_{\text{phan}}(r)$ is unity) at all distances (1–15 cm) along the transverse axis of the sources. “No” implies that the phantoms show distance‐dependent $k_{\text{phan}}(r)$ values (figure number is shown in parenthesis).

	*Diamond / Plastic Scintillator*		$Al_{2}O_{3}$		$Li_{2}B_{4}O_{7}$		*LiF*	
*Phantom Materials*	${\;}^{60}\text{Co}$	${\;}^{192}\text{Ir}$	${\;}^{60}\text{Co}$	${\;}^{192}\text{Ir}$	${\;}^{60}\text{Co}$	${\;}^{192}\text{Ir}$	${\;}^{60}\text{Co}$	${\;}^{192}\text{Ir}$
PMMA	No (Fig. 2)	No (Fig.5)	Ye s	No (Fig.5)	No (Fig.2)	No (Fig.5)	No (Fig.2)	Ye s
Polystyrene	Yes	No (Fig.6)	No (Fig.1)	No (Fig.6)	Yes	No (Fig.6)	Yes	No (Fig.6)
Plastic water	Yes	No (Fig.8)	No (Fig.1)	No (Fig.8)	Yes	No (Fig.8)	Yes	No (Fig.8)
RW1	Yes	No (Fig.7)	Yes	No (Fig.7)	Yes	No (Fig.7)	Yes	No (Fig.7)
RW3	Yes	Yes	Yes	No (Fig.4)	Yes	Yes	Yes	Yes
Virtual water	Yes	Yes	Yes	No (Fig.4)	Yes	Yes	Yes	Yes
Solid water	Yes	Yes	Yes	No (Fig.4)	Yes	Yes	Yes	Yes
A150	No (Fig. 3)	No (Fig.9)	No (Fig.3)	No (Fig.9)	No (Fig.3)	No (Fig.9)	No (Fig.3)	No (Fig.9)
WE210	Yes	Yes	Yes	No (Fig.4)	Yes	Yes	Yes	Yes

#### C.1 ${\;}^{60}\text{Co}$ source

**Figure 1:**
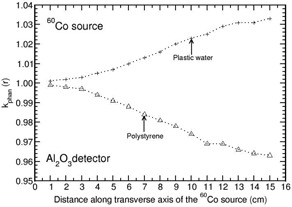
Phantom scatter correction, $k_{\text{phan}}(r)$, presented for ${\text{Al}}_{2}O_{3}$ detector in polystyrene and plastic water phantoms as a function of distance along the transverse axis of the BEBIG ${\;}^{60}\text{Co}$ brachytherapy source.

**Figure 2:**
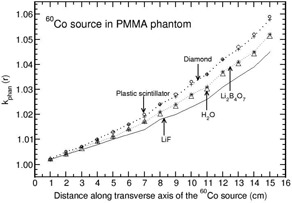
Phantom scatter correction, $k_{\text{phan}}(r)$, presented for PMMA phantom as a function of distance along the transverse axis of the BEBIG ${\;}^{60}\text{Co}$ brachytherapy source. The values are presented for detector materials LiF, ${\text{Li}}_{2}B_{4}O_{7}$, diamond, plastic scintillator, and water.

**Figure 3:**
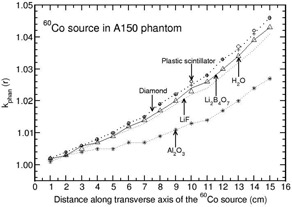
Phantom scatter correction, $k_{\text{phan}}(r)$, presented for A150 phantom as a function of distance along the transverse axis of the BEBIG ${\;}^{60}\text{Co}$ brachytherapy source. The values are presented for detector materials LiF, ${\text{Li}}_{2}B_{4}O_{7}$, diamond, plastic scintillator, ${\text{Al}}_{2}O_{3}$, and water.

Phantoms such as solid water, virtual water, RW1, RW3, and WE210 are water‐equivalent (i.e., $k_{\text{phan}}(r)$ is unity) at all distances (1–15 cm) for all the solid‐state detectors (maximum deviation from unity is about 1% at 15 cm for ${\text{Al}}_{2}O_{3}$ detector in solid water, RW1, and RW3). Polystyrene and plastic water phantoms are water‐equivalent at all distances for all the detectors (with a maximum deviation of about 1% from unity for LiF), other than ${\text{Al}}_{2}O_{3}$. Figure 1 presents the distance‐dependent $k_{\text{phan}}(r)$ values for the ${\text{Al}}_{2}O_{3}$ detector in plastic water and polystyrene phantoms. PMMA is water‐equivalent at all distances for ${\text{Al}}_{2}O_{3}$ detector (larger than unity by about 1% at 15 cm), whereas $k_{\text{phan}}(r)$ increases with r for remaining detector materials, including water (see Fig. 2). In this phantom, $k_{\text{phan}}(r)$ values are comparable for diamond, plastic scintillator, ${\text{Li}}_{2}B_{4}O_{7}$, LiF, and water detectors at all distances. For A150 phantom, $k_{\text{phan}}(r)$ increases with r for all the detectors, including water (see Fig. 3). For this phantom, $k_{\text{phan}}(r)$ values are comparable for the detectors diamond, plastic scintillator, ${\text{Li}}_{2}B_{4}O_{7}$, LiF. and water at all distances, with a maximum value of about 1.045 at 15 cm. For ${\text{Al}}_{2}O_{3}$, the maximum value of $k_{\text{phan}}(r)$ is 1.027 at 15 cm.

**Figure 4:**
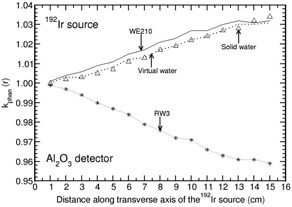
Phantom scatter correction, $k_{\text{phan}}(r)$, presented for ${\text{Al}}_{2}O_{3}$ detector in virtual water, solid water, RW3, and WE210 phantoms as a function of distance along the transverse axis of the ${\;}^{192}\text{Ir}$ brachytherapy source.

**Figure 5:**
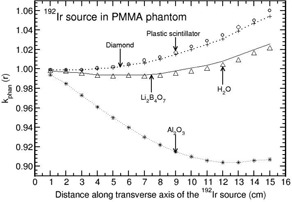
Phantom scatter correction, $k_{\text{phan}}(r)$, presented for PMMA phantom as a function of distance along the transverse axis of the ${\;}^{192}\text{Ir}$ brachytherapy source. The values are presented for detector materials ${\text{Li}}_{2}B_{4}O_{7}$, diamond, plastic scintillator, ${\text{Al}}_{2}O_{3}$, and water.

**Figure 6:**
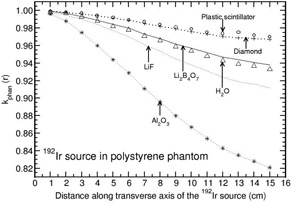
Phantom scatter correction, $k_{\text{phan}}(r)$, presented for polystyrene phantom as a function of distance along the transverse axis of the ${\;}^{192}\text{Ir}$ brachytherapy source. The values are presented for detector materials ${\text{Li}}_{2}B_{4}O_{7}$, LiF, diamond, plastic scintillator, ${\text{Al}}_{2}O_{3}$, and water.

**Figure 7:**
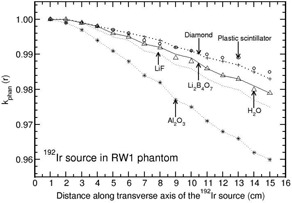
Same as Fig. 6, but for RW1 phantom.

**Figure 8:**
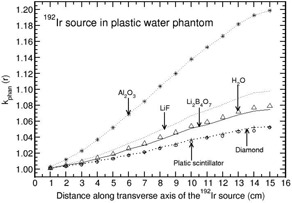
Same as Fig. 6, but for plastic water phantom.

**Figure 9:**
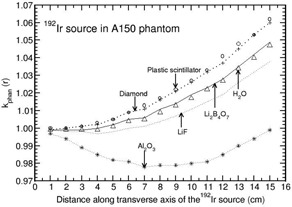
Same as Fig. 6, but for A150 phantom.

#### C.2 ${\;}^{192}\text{Ir}$ source

Phantoms such as solid water, virtual water, RW3, and WE210 are water‐equivalent in the distance range of 1–15 cm for all the detectors other than ${\text{Al}}_{2}O_{3}$ (with a maximum deviation of about 2% at 15 cm for solid water and RW3 phantoms). Figure 4 presents the distance‐dependent $k_{\text{phan}}(r)$ values of ${\text{Al}}_{2}O_{3}$ detector for the above four phantom materials. For this detector, $k_{\text{phan}}(r)$ increases with r for solid water, virtual water, and WE210 phantoms and decreases with r for RW3 phantom. $k_{\text{phan}}(r)$ is comparable for solid water, virtual water, and WE210 phantoms.

PMMA is water‐equivalent for LiF detector. Figure 5 presents $k_{\text{phan}}(r)$ values for all the detector materials other than LiF. For this phantom, $k_{\text{phan}}(r)$ decreases with r for ${\text{Al}}_{2}O_{3}$ detector (about 10% at 15 cm), whereas $k_{\text{phan}}(r)$ increases with r for all the other detectors. The degree of increase is higher for diamond detector and plastic scintillator (maximum deviation from unity at 15 cm is about 5% and 6%, respectively).

The phantoms polystyrene, RW1, plastic water, and A150 show distance‐dependent $k_{\text{phan}}(r)$ values which are presented in Figs. 6 to 9. $k_{\text{phan}}(r)$ decreases with r for all the detector materials in polystyrene and RW1 phantoms (Figs. 6 and 7). However, the degree of decrease is higher for ${\text{Al}}_{2}O_{3}$ detector compared to all other detectors. For example, the value decreases to 0.821 and 0.960 at 15 cm for polystyrene and RW1 phantoms, respectively. For plastic water phantom, $k_{\text{phan}}(r)$ values increase with r for all the detector materials, including water (Fig. 8). The degree of increase is higher for ${\text{Al}}_{2}O_{3}$ detector (about 20% larger than unity at 15 cm) compared to all other detectors (minimum deviation of about 5% from unity at 15 cm for diamond and plastic scintillator detector).

**Figure 10:**
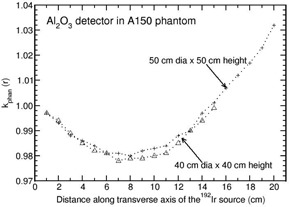
Phantom scatter correction, $k_{\text{phan}}(r)$, presented for ${\text{Al}}_{2}O_{3}$ detector in 40cm×40cm and 50cm×50cm A150 phantoms. The calculations are based on the FLURZnrc user‐code.

In the case of A150 phantom, $k_{\text{phan}}(r)$ value increases with r for all the detector materials (maximum deviation of about 6% from unity at 15 cm for diamond detector) other than ${\text{Al}}_{2}O_{3}$ detector (Fig. 9). For ${\text{Al}}_{2}O_{3}$ detector, $k_{\text{phan}}(r)$ decreases from 0.997 (at 1 cm) to 0.978 (at 7 cm) and thereafter increases to unity at a distance of 15 cm. In order to verify this trend beyond 15 cm, auxiliary simulations are carried out using the FLURZnrc user‐code^(11)^ with larger dimensions (50 cm diameter ×50cm height) of A150 and water phantoms, to calculate $k_{\text{phan}}(r)$ for r=1–20cm. Figure 10 compares $k_{\text{phan}}(r)$ values obtained in 50 cm diameter ×50cm height and 40 cm diameter ×40cm height phantoms for ${\text{Al}}_{2}O_{3}$ detector. Up to 15 cm $k_{\text{phan}}(r)$ values are comparable in both the phantom dimensions. For 50 cm diameter ×50cm height phantom, $k_{\text{phan}}(r)$ reaches the value of 1.032 at r=20cm. To verify any possible influence of the detector dimensions on $k_{\text{phan}}(r)$, separate auxiliary simulations are also carried out with 50 cm diameter ×50cm height phantom by using the DOSRZnrc user‐code,^(10)^ in which ${\text{Al}}_{2}O_{3}$ detector is modeled as a 1 mm thick ×2mm high cylinder. The values of $k_{\text{phan}}(r)$ are calculated along the transverse axis of the ${\;}^{192}\text{Ir}$ source for r=1,5,10,15, and 20 cm. The study shows that DOSRZnrc‐based $k_{\text{phan}}(r)$ values are statistically identical to the corresponding FLURZnrc‐based $k_{\text{phan}}(r)$ values.

### D. Influence of detector dimensions on detector response

The above‐described FLURZnrc‐based calculated values of $k_{\text{phan}}(r)$ and $k_{\text{phan}}(r)$ are based on the assumption that charged particle equilibrium exists and the presence of detector does not affect the above corrections. In order to quantify the influence of detector thicknesses on the calculated response, auxiliary simulations are carried out in water phantom using the DOSRZnrc user‐code.^(10)^ LiF, ${\text{Li}}_{2}B_{4}O_{7}$, plastic scintillator, and ${\text{Al}}_{2}O_{3}$ detectors are modeled as cylindrical shells of thickness 1 mm and height 2 mm, whereas diamond detector is modeled for two different thicknesses (0.2 mm and 0.4 mm) and height 2 mm. Absorbed dose and collision kerma to these detectors are calculated at r=1 and 15 cm. For ${\;}^{192}\text{Ir}$ source, collision kerma and absorbed‐dose values are statistically identical for all the detectors. For ${\;}^{60}\text{Co}$ source, collision kerma and absorbed‐dose values are statistically identical for ${\text{Al}}_{2}O_{3}$, plastic scintillator, ${\text{Li}}_{2}B_{4}O_{7}$, and LiF detectors. In the case of diamond detector, the absorbed dose values are smaller by about 1% at 1 cm and about 1.5% at 15 cm, compared to the collision kerma values.

## IV. CONCLUSIONS

Beam quality correction, $k_{{\text{QQ}}_{0}}(r)$, for solid‐state detector materials such as diamond, plastic scintillator, LiF, ${\text{Li}}_{2}B_{4}O_{7}$, and ${\text{Al}}_{2}O_{3}$ are calculated as a function of distance along the transverse axis of the ${\;}^{60}\text{Co}$ and ${\;}^{192}\text{Ir}$ brachytherapy sources using the Monte Carlo‐based EGSnrc code system. For ${\;}^{60}\text{Co}$ source, $k_{{\text{QQ}}_{0}}(r)$ is about unity and distance independent for diamond, plastic scintillator, ${\text{Li}}_{2}B_{4}O_{7}$, and LiF detector, and decreases gradually with r for ${\text{Al}}_{2}O_{3}$ (about 6% lesser than unity at 15 cm). For ${\;}^{192}\text{Ir}$ source, $k_{{\text{QQ}}_{0}}(r)$ is about unity and independent of distance for ${\text{Li}}_{2}B_{4}O_{7}$ detector. $k_{{\text{QQ}}_{0}}(r)$ increases with distance for diamond and plastic scintillator (about 6% and 8% larger than unity at 15 cm, respectively). $k_{{\text{QQ}}_{0}}(r)$ decreases gradually with r for LiF and steeply for ${\text{Al}}_{2}O_{3}$.

Phantom scatter correction, $k_{\text{phan}}(r)$, for various solid phantoms are calculated for the above detectors along the transverse axis of ${\;}^{60}\text{Co}$ and ${\;}^{192}\text{Ir}$ sources. For ${\;}^{60}\text{Co}$ source, phantoms such as solid water, virtual water, RW1, RW3, and WE210 are water‐equivalent for all the investigated detectors. Polystyrene and plastic water phantoms are water‐equivalent for diamond, plastic scintillator, ${\text{Li}}_{2}B_{4}O_{7}$, and LiF detectors, but shows distance‐dependent $k_{\text{phan}}(r)$ values for ${\text{Al}}_{2}O_{3}$ detector. PMMA is water‐equivalent at all distances for ${\text{Al}}_{2}O_{3}$, but shows distance‐dependent $k_{\text{phan}}(r)$ values for remaining detectors. A150 phantom shows distance‐dependent $k_{\text{phan}}(r)$ values for all the detector materials. For ${\;}^{192}\text{Ir}$ source, solid water, virtual water, RW3, and WE210 phantoms are water‐equivalent for diamond, plastic scintillator, ${\text{Li}}_{2}B_{4}O_{7}$, and LiF detectors, whereas these phantoms show distance‐dependent $k_{\text{phan}}(r)$ values for ${\text{Al}}_{2}O_{3}$ detector. Remaining phantom materials demonstrated distance‐dependent $k_{\text{phan}}(r)$ values, but the degree of dependence depends on the type of solid phantom and the detector. ${\text{Li}}_{2}B_{4}O_{7}$ detector shows $k_{\text{phan}}(r)$ values identical to that of water detector, and diamond detector shows $k_{\text{phan}}(r)$ values identical to that of plastic scintillator detector for all the investigated phantoms for ${\;}^{192}\text{Ir}$ and ${\;}^{60}\text{Co}$ sources.

## ACKNOWLEDGMENTS

The authors would like to thank Dr. D. N. Sharma, Director, Health, Safety & Environment Group, Bhabha Atomic Research Centre (BARC), and Mr. D. A. R. Babu, Head, Radiological Physics & Advisory Division, BARC for their encouragement and support throughout the study.
